# Fatal COVID‐19 infection in a patient with long‐chain 3‐hydroxyacyl‐CoA dehydrogenase deficiency: A case report

**DOI:** 10.1002/jmd2.12165

**Published:** 2020-09-10

**Authors:** Parith Wongkittichote, James R. Watson, Jennifer M. Leonard, Elizabeth R. Toolan, Patricia I. Dickson, Dorothy K. Grange

**Affiliations:** ^1^ Division of Genetics and Genomic Medicine, Department of Pediatrics Washington University School of Medicine St Louis Missouri USA; ^2^ Division of Hospital Medicine, Department of Medicine Washington University School of Medicine St Louis Missouri USA; ^3^ Department of Surgery Washington University School of Medicine St Louis Missouri USA

**Keywords:** cardiomyopathy, COVID‐19, fatty acid oxidation disorder, LCHAD, long‐chain 3‐hydroxyacyl‐CoA dehydrogenase deficiency

## Abstract

Long‐chain fatty‐acyl CoA dehydrogenase deficiency (LCHADD) is an inborn error of long chain fatty acid oxidation with various features including hypoketotic hypoglycemia, recurrent rhabdomyolysis, pigmentary retinopathy, peripheral neuropathy, cardiomyopathy, and arrhythmias. Various stresses trigger metabolic decompensation. Coronavirus disease 2019 (COVID‐19) is a pandemic caused by the RNA virus SARS‐CoV‐2 with diverse presentations ranging from respiratory symptoms to myocarditis. We report a case of a patient with LCHADD who initially presented with typical metabolic decompensation symptoms including nausea, vomiting, and rhabdomyolysis in addition to mild cough, and was found to have COVID‐19. She developed acute respiratory failure and refractory hypotension from severe cardiomyopathy which progressed to multiple organ failure and death. Our case illustrates the need for close monitoring of cardiac function in patients with a long‐chain fatty acid oxidation disorder.

**Synposis**

Patients with inborn errors of metabolism should be considered a vulnerable population for COVID‐19, given the risks of severe complications.

## 1 INTRODUCTION

Long‐chain fatty‐acyl CoA dehydrogenase deficiency (LCHADD, OMIM #609016) is an inborn error of metabolism caused by pathogenic variants in *HADHA*.^1^ Mitochondrial trifunctional protein (MTP) consists of four alpha subunits encoded by *HADHA* and four beta subunits encoded by *HADHB*.^2^ MTP catalyzes three steps in long chain fatty acid oxidation including long‐chain 3‐hydroxyacyl‐CoA dehydrogenase (LCHAD), long‐chain enoyl‐CoA hydratase (LCEH), and long‐chain 3‐ketoacyl‐CoA thiolase (LCKAT).^3^, ^4^ Alpha subunits possess LCHAD and LCEH activities, while beta subunits have LCKAT activity.^5^ Pathogenic variants in *HADHA* disrupting LCHAD activity cause LCHADD,^6^ while those in *HADHA* and *HADHB* causing loss of function in other enzymatic activities of MTP leads to trifunctional protein deficiency (mTPD, OMIM #609015).^7^, ^8^, ^9^ Clinical characteristics of patients with LCHADD and mTPD are similar to other long chain fatty acid oxidation disorders, including hypoketotic hypoglycemia, recurrent rhabdomyolysis, cardiomyopathy, and arrhythmias^10^, ^11^, ^12^, ^13^, ^14^, ^15^; however, LCHADD and mTPD can cause peripheral neuropathy and pigmentary retinopathy that are irreversible.^12^, ^13^, ^14^, ^15^, ^16^, ^17^ Age of onset and severity of symptoms of LCHADD and mTPD are highly variable.^12^ Physiologic stress, including illness, and inappropriate diet cause acute metabolic decompensation and may lead to death.^18^ Dietary treatment with long chain fatty acid restriction and medium chain triglyceride (MCT) supplementation are the mainstays of LCHADD treatment and prevention of complications.^19^

Coronavirus disease 2019 (COVID‐19) is an emerging disease caused by a novel coronavirus, namely severe acute respiratory syndrome coronavirus 2 (SARS‐CoV‐2).^20^ COVID‐19 was declared a pandemic on 11 March 2020 by the World Health Organization.^21^ The most common presenting symptoms of COVID‐19 include fever, fatigue, dry cough, myalgia, dyspnea, and loss of olfactory sensation.^22^, ^23^ Headache, dizziness, abdominal pain, diarrhea, nausea, and vomiting are less common presenting symptoms.^22^, ^23^ Acute respiratory distress syndrome and sepsis are major complications associated with death in COVID‐19 patients.^24^ Cardiovascular involvement, including cardiomyopathy, ventricular arrhythmia, and hemodynamic instability have been reported in COVID‐19 patients without underlying cardiovascular disease.^25^, ^26^

Infections are common triggers of metabolic decompensation in patients with inborn errors of metabolism, leading to a higher risk for severe complications. It is unknown whether individuals with inborn errors of metabolism are at greater risk of serious illness due to COVID‐19. Here we report a patient with LCHADD and COVID‐19 who developed severe cardiomyopathy and multiple organ system failure leading to death.

## 2 CASE REPORT

The patient was a 23‐year‐old Caucasian female who presented to a local hospital due to chest pain and nausea. Three days prior to admission, she developed an intermittent dry cough. One day prior to admission, she developed chest and epigastric pain as well as nausea and vomiting similar to her prior episodes of metabolic decompensation and rhabdomyolysis. She had a decrease in oral intake. At the local hospital she was found to have an elevated creatine kinase level of 15 380 units/mL (normal range 30‐200 units/L) along with an elevated troponin T level of 0.23 ng/mL (normal range 0.00‐0.03 ng/mL), and a creatinine of 0.49 mg/dL (normal range 0.60‐1.10 mg/dL). Her vital signs were normal and she did not require oxygen supplementation. She was transported to our hospital for management. On arrival, she tested positive for COVID‐19 by nasopharyngeal swab real‐time RT‐PCR assay and was transferred to a COVID‐19 isolation unit.

Her past medical history was notable for LCHADD which was diagnosed at 4 months of age when she presented with apnea, lactic acidosis, and hypoglycemia. Her mother's pregnancy had been complicated by acute fatty liver of pregnancy. Genetic testing revealed compound heterozygosity for a paternally inherited c.274_278del (p.Ser92fs) variant and a maternally inherited c.1528G>C (p.Glu510Gln) variant. Lactic acidosis and hypoglycemia in infancy resolved after dietary treatment for LCHADD was instituted. She had multiple complications of LCHADD including progressive retinopathy with severe visual impairment and peripheral neuropathy. During pregnancy at age 21, she was diagnosed with long QT interval with self‐limited torsade de pointes, and was treated with propranolol. Her last echocardiogram 6 weeks prior to this hospitalization showed a normal left ventricular ejection fraction (LVEF) of 62% and normal right ventricular function. Regarding LCHADD management, she was prescribed 44 g/d of medium‐chain triglyceride and l‐carnitine supplementation with a low‐fat dietary restriction. Intermittent non‐compliance had led to multiple episodes of metabolic decompensation and rhabdomyolysis.

On arrival to our hospital, she continued to experience nausea, vomiting, and intermittent dry cough. Telemetry monitoring was initiated. She was started on deep vein thrombosis prophylaxis with 40 mg of subcutaneous enoxaparin daily. Her electrocardiogram was notable only for a prolonged QT interval of 526 ms which was unchanged from prior studies. She initially received an intravenous fluid bolus with 1 L of Ringer's lactate solution and was transitioned to continuous intravenous fluids containing 10% dextrose at 1.5 times maintenance rate (150 mL/h) for a glucose infusion rate (GIR) of 3.4 mg/kg/min. Her intake of MCTs was increased to 70 g/day with continuation of l‐carnitine supplement and dietary fat intake restriction. She was instructed to drink Ensure Clear for additional calories and protein. However, she was not able to achieve her nutritional goal due to nausea, leading to further elevation in her creatine kinase to 40 600 units/L on day 4 of admission. She developed acute kidney injury with creatinine rising from 0.51 mg/dL on admission to 2.25 mg/dL despite the increase of intravenous fluids containing 10% dextrose to a GIR of 4.2 mg/kg/min. She began to show signs of volume overload with peripheral edema. The patient did not tolerate placement of a nasogastric tube for feeding. A PICC line was placed on day 2 of admission and the patient was started on parenteral nutrition supplementation on day 5 of admission with dextrose 480 g/d and protein 1.5 g/kg/d. By day 11 of admission, her creatine kinase and serum creatinine improved to 1669 units/L and 0.72 mg/dL, respectively. She continued to have an intermittent cough, but had no shortness of breath or requirement for supplemental oxygen. Her vital signs were stable with the exception of an intermittent fever, and review of her telemetry showed no events.

On day 11 of admission, she developed acute onset of oxygen desaturation. On arrival to the bedside, nursing noted that the patient was confused and intermittently voicing difficult breathing, in visible respiratory distress, and ashen with cold extremities. She was afebrile. They were unable to obtain reliable oxygen saturation or blood pressure. The rapid response team was called. Anesthesia performed rapid sequence intubation with video‐assisted laryngoscopy. She was placed on pressure‐regulated volume control mechanical ventilation. Her arterial blood gas after intubation revealed a pH 7.23, pCO_2_ 33 mm Hg, pO_2_ 262 mm Hg, base excess −13. Her lactate was 12.8 mmol/L (reference range 0.7‐2.0 mmol/L). N‐terminal pro‐brain natriuretic peptide was 902 pg/mL (normal range < 300 pg/mL), compared to a level of 1205 pg/mL on day 6 of admission. Fibrinogen was 816 mg/dL (normal range 170‐400 mg/dL). D‐dimer was 1467 ng/mL (normal range < 499 ng/mL). Troponin I was 0.08 ng/mL (0.00‐0.03 ng/mL). No blood gas was obtained prior to intubation. She was profoundly hypotensive after intubation with systolic blood pressures of 30‐40 mm Hg. She was started on pressor support with a norepinephrine drip and transferred to the ICU.

On arrival to the ICU, bedside cardiac and lower extremity ultrasound revealed decreased right ventricular function and suspected lower extremity venous clots. She was given 200 mg of alteplase empirically due to concern for pulmonary embolism causing obstructive shock and hypoxemic respiratory failure. She was started on empiric broad spectrum antibiotics with linezolid, cefepime, and micafungin. Despite these interventions, the patient required rapidly increasing levels of pressor support with the addition of vasopressin and epinephrine. A few hours after transfer to the ICU, venoarterial extracorporeal membrane oxygenation (VA‐ECMO) was initiated with cannulas in the right common femoral vein and left common femoral artery. Despite multiple attempts, the cardiothoracic surgery team was unable to visualize or access the left superficial femoral artery for placement of a distal perfusion catheter.

A computed tomography (CT) of the chest 18 hours after alteplase did not show a PE but was notable for small bilateral pleural effusions with right lower lobe and partial left lower lobe collapse, scattered ground glass opacities, and septal line thickening. Transthoracic echocardiogram revealed moderate global left ventricular dysfunction with an LVEF of 34% and moderate right ventricular dilatation with severe right ventricular hypokinesis. Review of her telemetry showed no evidence of arrhythmia or ischemia during the peri‐decompensation period. Urinalysis revealed leukocyte esterase 3+, protein 2+, nitrite negative, bacteria 3+, greater than 50 white blood cells per high power field, and greater than 50 red blood cells per high power field. Urine culture was positive for *Escherichia coli*. Blood cultures grew *Enterobacter cloacae*. The source of her bacteremia was thought to be related either to her PICC line or to gut translocation. Her PICC line was removed and parenteral nutrition was stopped. She was diagnosed with combined cardiogenic and septic shock. She did not receive systemic corticosteroids.

After ECMO cannulation, she had undetectable dorsalis pedis pulses by palpation and arterial Doppler ultrasound. Vascular surgery believed that the combination of severe hypotension and ECMO placement had led to poor perfusion of her lower extremities, but no surgical intervention was immediately indicated. On day 12 of admission, liver function studies showed newly elevated transaminases with an alanine aminotransferase of 800 units/L (normal range 7‐45 units/L) and an aspartate aminotransferase of 1175 units/L (normal range 10‐45 units/L). Her ammonia level was normal at 48 μmol/L. Elevated transaminases were attributed to ischemic liver injury. Despite continuing to be critically ill on ventilator and pressor support, her condition stabilized for a few days after ECMO cannulation. Notably, her kidney function remained normal with good urine output and her creatinine kinase levels remained stable at 3000‐4000 units/L despite the cessation of parenteral nutrition. A repeat echocardiogram on day 13 demonstrated RV dilation with normalization of RV systolic function and improvement of LVEF to 41%. On day 15, she had increased mottling of the left foot and therefore, she underwent ECMO revision. Post‐operatively, she developed worsening hypotension and lactic acidosis. A CT of the chest, abdomen, and pelvis on day 16 revealed ischemic nonocclusive enteritis involving the majority of the small bowel and ascending colon with suspicion for mucosal breach in the right lateral wall of the descending colon. Emphysematous esophagitis and gastritis were also suspected on the CT. Despite aggressive measures, she developed sustained refractory hypotension leading to a decision to withdraw medical support. She died on day 17 of admission. An autopsy was not performed due to COVID‐19.

## 3 DISCUSSION

We report a patient with LCHADD admitted due to COVID‐19 and rhabdomyolysis. She initially presented with mild upper respiratory tract symptoms and did not require oxygen supplementation. Metabolic decompensation led to rhabdomyolysis and acute kidney injury. Improvement in metabolic status and renal function were observed over the first week of hospitalization with adequate nutritional supplementation. However, she suddenly developed acute respiratory failure and acute cardiomyopathy, leading to death despite maximal support.

Cardiomyopathy is a major complication of long‐chain fatty acid oxidation disorders including LCHADD and mTPD.^10^, ^11^, ^12^, ^13^ Patients with LCHADD and mTPD can develop hypertrophic cardiomyopathy, dilated cardiomyopathy, and arrhythmias.^27^, ^28^ The pathophysiology of cardiac complications in LCHADD is still unclear. Proposed mechanisms include the accumulation of toxic long‐chain acylcarnitines or deficiency of energy substrate.^28^, ^29^ Severe, acute cardiomyopathy with rapid reduction in LVEF has been reported in an LCHADD patient with an acute metabolic crisis, exacerbated by dietary noncompliance and infection,^27^ similar to our patient whose echocardiogram was normal prior to this admission.

COVID‐19 is an emerging infectious disease that became a global public health emergency and pandemic.^21^ Although COVID‐19 was initially thought to primarily involve the respiratory tract, recent studies have revealed extrapulmonary manifestations. Cardiac involvement has been reported in multiple cases and cardiomyopathy is the most common cardiac complication.^22^, ^23^, ^24^, ^25^, ^30^ Another report described a patient who presented with cardiomyopathy with minimal respiratory symptoms, similar to our patient.^25^ Recent studies revealed that approximately one‐third of the patients with severe COVID‐19 requiring intensive care exhibited cardiomyopathy.^31^ Those patients who have underlying cardiac disease are at risk of severe COVID‐19.^30^, ^32^ These studies illustrate the strong association of COVID‐19 with cardiac involvement. Several mechanisms leading to myocardial injury are proposed, including myocardial injury caused by viral infection, systemic inflammation leading to cytokine storm, altered myocardial metabolic demand, abnormal coagulation leading to thromboembolism, adverse effects from treatment, and electrolyte imbalance.^30^ In our patient, one possibility is that COVID‐19 and systemic inflammation led to increased metabolic demand and eventually metabolic crisis which caused LCHADD‐related cardiomyocyte injury in addition to viral myocarditis. We hypothesize that this vicious cycle eventually caused cardiomyopathy and cardiac dysfunction. Coagulation disorders are known to be relatively common complications of COVID‐19.^33^ A hypercoagulable state leading to thrombi formation that could not be detected by CT or were quickly cleared by fibrinolytics or anticoagulants is another possible cause of the acute deterioration seen in our patient. Autopsy with analysis of cardiac tissue could have provided more information and possible mechanisms leading to cardiomyopathy. However, due to COVID‐19 restrictions, autopsy was not performed.

In conclusion, we report a case of LCHADD patient with fatal COVID‐19. This case illustrates the need for close monitoring of cardiac function in patients with fatty acid oxidation defects who develop COVID‐19. Patients with inborn errors of metabolism should be considered a vulnerable population for COVID‐19, given the risks of developing metabolic decompensation and severe complications.

## CONFLICT OF INTEREST

The authors declare no conflicts of interest.

## AUTHOR CONTRIBUTIONS

Parith Wongkittichote was involved in drafting and revising manuscript. Dorothy K. Grange was involved in consent and revising manuscript. James R. Watson, Jennifer M. Leonard, Elizabeth R. Toolan, and Patricia I. Dickson were involved with revising manuscript.

## A PATIENT CONSENT STATEMENT

Informed consent was obtained from the patient's family for publication of this case report.

## ANIMAL RIGHTS

This article does not contain any studies with human or animal subjects performed by the any of the authors.
